# Process evaluation of a digital school-based intervention: lessons learned from the Health4Life study

**DOI:** 10.1093/tbm/ibag060

**Published:** 2026-09-14

**Authors:** Katrina E Champion, Lauren A Gardner, Bridie Osman, Siobhan O’Dean, Emily Hunter, Louise Thornton, Cath Chapman, Tim Slade, Nyanda McBride, Courtney Stewart, Matthew Sunderland, Leanne Hides, Steve Allsop, David R Lubans, Belinda Parmenter, Bonnie Spring, Lyra Egan, Scarlett Smout, Maree Teesson, Nicola C Newton

**Affiliations:** The Matilda Centre for Research in Mental Health and Substance Use, The University of Sydney, Sydney, NSW, Australia; Sydney School of Public Health, The University of Sydney, Sydney, NSW, Australia; The Matilda Centre for Research in Mental Health and Substance Use, The University of Sydney, Sydney, NSW, Australia; The Matilda Centre for Research in Mental Health and Substance Use, The University of Sydney, Sydney, NSW, Australia; The Matilda Centre for Research in Mental Health and Substance Use, The University of Sydney, Sydney, NSW, Australia; The Matilda Centre for Research in Mental Health and Substance Use, The University of Sydney, Sydney, NSW, Australia; The Matilda Centre for Research in Mental Health and Substance Use, The University of Sydney, Sydney, NSW, Australia; Hunter Medical Research Institute, New Lambton Heights, NSW, Australia; School of Medicine and Public Health, The University of Newcastle, Callaghan, NSW, Australia; The Matilda Centre for Research in Mental Health and Substance Use, The University of Sydney, Sydney, NSW, Australia; The Matilda Centre for Research in Mental Health and Substance Use, The University of Sydney, Sydney, NSW, Australia; National Drug Research Institute, enAble Institute, Faculty of Health Science, Curtin University, Perth, WA, Australia; National Drug Research Institute, enAble Institute, Faculty of Health Science, Curtin University, Perth, WA, Australia; The Matilda Centre for Research in Mental Health and Substance Use, The University of Sydney, Sydney, NSW, Australia; School of Psychology, The University of Queensland, St Lucia, QLD, Australia; National Centre for Youth Substance Use Research, The University of Queensland, QLD, Australia; National Drug Research Institute, enAble Institute, Faculty of Health Science, Curtin University, Perth, WA, Australia; Hunter Medical Research Institute, New Lambton Heights, NSW, Australia; Global Sport and Movement Collaborative, University of Newcastle, Callaghan, NSW, Australia; Faculty of Sport and Health Sciences, University of Jyväskylä, Jyväskylä, Finland; School of Health Sciences, University of New South Wales, Sydney, NSW, Australia; School of Health, University of the Sunshine Coast, Sippy Downs, QLD, Australia; Department of Preventive Medicine, Northwestern University Feinberg School of Medicine, Chicago, IL, United States; Department of Behavioral Sciences and Social Medicine, Florida State University College of Medicine, Tallahassee, FL, United States; The Matilda Centre for Research in Mental Health and Substance Use, The University of Sydney, Sydney, NSW, Australia; The Matilda Centre for Research in Mental Health and Substance Use, The University of Sydney, Sydney, NSW, Australia; The Matilda Centre for Research in Mental Health and Substance Use, The University of Sydney, Sydney, NSW, Australia; The Matilda Centre for Research in Mental Health and Substance Use, The University of Sydney, Sydney, NSW, Australia

**Keywords:** prevention, school-based, intervention, implementation, risk behaviors

## Abstract

**Background:**

Process evaluations of preventive health interventions are essential for enhancing their implementation and impact.

**Purpose:**

This study presents the process evaluation of *Health4Life*, a digital school-based program designed to modify multiple health risk behaviors among adolescents.

**Methods:**

Data were derived from a cluster randomized controlled trial in 71 Australian secondary schools from 2019 to 2022, including data from 2085 students and 118 teachers. Based on the Medical Research Council guidance for process evaluations this study explored three key questions: (i) Was the Health4Life intervention delivered and received as intended? (ii) How did participants respond? and (iii) What contextual factors influenced implementation? Fidelity, dose and exposure to intervention components were assessed through teacher self-report and website analytics. Participant responsiveness included student and teacher acceptability, and factors associated with a positive program evaluation (i.e. students’ ratings of enjoyment, relevance, and utility). Contextual factors relating to schools and the intervention were assessed via teacher-reported open-ended feedback. The Consolidated Framework for Implementation Research (CFIR) was applied to support interpretation of findings.

**Results:**

Fidelity to the online modules was high based on teacher self-report, but the dose received by students was moderate (62% full dose), and use of the supplementary components varied. Program acceptability was high, with most students (73%) and teachers (84%) rating *Health4Life* favorably overall. Key factors associated with a positive student evaluation were identifying as female, and having higher baseline knowledge of health risk behaviors, greater self-control and higher socioeconomic advantage. Qualitative data identified key contextual barriers and facilitators relating to program design, technology and school contextual issues.

**Conclusions:**

These findings provide valuable insights to support the ongoing refinement and broader implementation of *Health4Life*. A set of recommendations are proposed to inform the future development, implementation, and evaluation of digital health interventions in school environments.

**Clinical Trial information:**

The *Health4Life* study was pre-registered with the Australian New Zealand Clinical Trials Registry (ACTRN12619000431123).

## Teaser

- A digital school program to support healthy habits was delivered consistently by teachers and viewed positively by most high school students.
- Findings highlight the need for flexible delivery and tailored content to better engage students from diverse backgrounds.

Implications **Practice:** Digital school-based health interventions should incorporate flexible delivery modes and tailored content to student knowledge level, gender, and socioeconomic context to maximize engagement and equitable reach.

**Policy:** Education and health systems should invest in digital infrastructure, implementation support, and codesign processes that enable schools to deliver adaptable, inclusive digital prevention programs at scale.

**Research:** Further research should incorporate multi-method process evaluations that examine teacher training and engagement, school context, and differential student responses to inform adaptive, context‑responsive digital school‑based interventions.

High-quality implementation is critical to the success of health promotion and prevention programs and is associated with improved participant outcomes [1, 2]. Process evaluations systematically assess the implementation of an intervention and can inform strategies to optimize content and delivery and enhance future impact [3, 4]. Process evaluations of school-based interventions are particularly important due to the diverse contexts and complex nature of the school environment. Schools involve multiple stakeholders, including teachers, students, parents and administrators, whose engagement with, and acceptance of, an intervention can directly affect its success [5]. In addition, schools face unique barriers (e.g. lack of time, competing priorities, available resources), and facilitators (e.g. teacher motivation, school support) to the successful implementation of an intervention [6–8]. Process evaluations can help to ensure that school-based programs are implemented efficiently and effectively, can be scaled, and are adaptable to diverse school contexts.

One school-based intervention is the *Health4Life Initiative,* a digital multiple health behavior change program designed to concurrently target six key health risk behaviors among adolescents: alcohol use, tobacco smoking, physical inactivity, poor diet, poor sleep, and excessive recreational screen time (“the Big 6”). *Health4Life* comprises a school-based program [9], which consists of six web-based modules and optional classroom activities delivered during health education lessons in the first year of secondary school (students aged 11–13). It was supplemented by an accompanying smartphone app [10], however this was only used by 11% of eligible students [11]. A cluster RCT in 71 Australian schools (2019–2022) showed that *Health4Life* did not modify the Big 6 risk behaviors (primary outcomes), when compared to an active control (health education as usual). However, it significantly improved secondary outcomes, including students’ knowledge about the Big 6, with effects lasting for two years [12], small and short-term improvements in depression and psychological distress [13], reduced intentions to try alcohol and tobacco, and increased intentions to improve sleep duration and reduce screen time [14].

Building on these findings, the present study aimed to explore the implementation of the *Health4Life* school-based program. The Medical Research Council (MRC) guidance [3] offers a framework for process evaluations of complex interventions within the context of a randomized controlled trial (RCT). Using MRC guidance, this study aimed to address the following research questions:

1. To what extent was the Health4Life intervention delivered and received as intended?
2. How did participants (students and teachers) respond?
3. What contextual factors influenced implementation?

Findings will be used to generate insights to optimize *Health4Life*, design strategies to enhance program implementation, and to develop a set of evidence-based recommendations to guide the future development, implementation, and evaluation of digital health interventions in schools.

## Methods

### Study design

This study uses mixed methods data collected as part of a cluster RCT. This process evaluation was guided by the MRC Process Evaluation Guidance [3], adapted to suit the school-based digital delivery context of *Health4Life* (see Table 1). The MRC guidance informed the overall design of the process evaluation, including the assessment of implementation, participant responsiveness, and contextual factors, while the Consolidated Framework for Implementation Research (CFIR) [15] was used as a complementary framework to interpret determinants of implementation across schools. The CFIR is a widely applied implementation science framework used to identify or explain barriers and facilitators to implementation success. Relevant CFIR domains included the *inner setting* (school-level contextual factors) and aspects of the *innovation* (i.e. intervention design and delivery format).

**Table 1:** Process evaluation outcome definitions and data sources.

MRC construct and research question	Sub-domain	Data source
**Implementation** To what extent was the intervention delivered and received as intended?	**Fidelity:** Adherence to the intended delivery of the Health4Life program.	Teacher self-report via logbook.
	**Dose:** Number of online student modules completed (sufficient vs insufficient dose); exposure to supplementary intervention materials, including student and teacher summaries, activities, and teacher implementation guide.	Website analytics, including number of opens, downloads, and clicks.
**Participant Responsiveness** How did participants (students and teachers) respond to the intervention?	**Student acceptability:** satisfaction, enjoyment, relevance, and utility of *Health4Life.*	Student self-report via a program evaluation survey, including quantitative and qualitative feedback.
	**Factors associated with a positive program evaluation:** factors linked to students’ satisfaction, enjoyment, relevance, and utility of *Health4Life.*	Student self-report via baseline survey, linked to their program evaluation survey.
	**Teacher acceptability:** satisfaction, ease of use and perceived usefulness to others of *Health4Life.*	Teacher self-report via a program evaluation survey, including quantitative and qualitative feedback.
**Context** **What contextual factors influenced implementation?**	**Barriers, facilitators and other external factors** affecting the implementation of *Health4Life.*	Teacher self-report via program evaluation survey and fidelity logbook.

### Participants and procedures

6639 students from 71 Australian secondary schools participated in the RCT, of which 3609 students (36 schools) were randomized to the *Health4Life* intervention and 3030 students (35 schools) to an active control. Full details of the trial have been published previously [12, 16]. A total of 210 teachers from the intervention group registered on the *Health4Life* website to assist in implementing the study. All students and teachers in the intervention group were automatically redirected to complete an online evaluation survey via the study website following completion of the final *Health4Life* lesson. Teachers were also asked to report on their implementation of *Health4Life* (via the study website or hard-copy) and were sent reminders to encourage weekly completion. Teachers were reimbursed $100AUD for their time. The study was approved by Human Research Ethics Committees of the University of Sydney (2018/882), the University of Queensland (2019000037), Curtin University (HRE2019-0083), and relevant school sector ethics committees.

### The Health4Life school-based program

*Health4Life* is a digital intervention comprising six online cartoon modules, supplemented by lesson summaries for teachers and students, web-based tailored feedback and optional online and teacher-delivered activities. The online cartoon modules are the core component of the intervention, intended to be delivered sequentially during health education (ideally once per week), which is a mandatory subject within the Australian curriculum for the first four years of secondary school. The cartoon modules use co-designed storylines about a group of teenagers with principles of social influence, social-cognitive, and self-determination theories, to impart evidence-based information about the Big 6. A detailed description of the module content and the intervention’s logic model has been published previously [9] (also see Supplementary Material). The cartoons were designed to be completed individually by students via the *Health4Life* Study website, under teacher supervision.

## Measures

### Intervention delivery

Implementation of the program (including fidelity, dose and exposure) was assessed via teacher self-report and objective website data. Fidelity (defined as adherence to the intended delivery of the intervention) was assessed using teacher logbooks which asked “Did your students complete the online *Health4life* cartoon component?” and “Did your students complete the whole online cartoon for module 1? If no, why not?” (see Supplementary Material for full logbook). Objective website data were used to assess dose of the intervention (number of online cartoon modules completed by students) and exposure to Supplementary Materials. Exposure was defined as teacher and student interaction with the Supplementary Materials (i.e. student and teacher activities, summaries, teacher implementation guide, and offline resources), assessed using objective indicators such as the number of opens, downloads, and clicks.

### Participant responsiveness

#### Teacher acceptability

Teachers answered 12-items assessing satisfaction, ease of use and perceived usefulness to others of *Health4Life*. For example, teachers were asked “How would you rate the *Health4Life* program in comparison to other school-based health education programs?” Responses were made on a five-point Likert scale (e.g. “much better” to “a lot worse”).

#### Student acceptability

Students were asked eight questions regarding satisfaction, relevance, and perceived utility of *Health4Life*. For example, “How much did you like learning in this way (i.e. via online cartoon modules)?,” “How relevant were the stories to experiences in your own life?,” and “How likely are you to use the skills and information taught in the program in your own life?”. Responses were made on a 5-point Likert scale (e.g. “Liked a lot” to “Disliked a lot”). Due to the skewed distribution of responses, with most students selecting positive options, variables were dichotomized (e.g. “Liked a little/Liked a lot” vs. “Neither liked nor disliked/Disliked a little/Disliked a lot”). This allowed for a clearer contrast between positive endorsement and neutral/negative responses.

#### Factors associated with a positive student evaluation

The following self-reported data were collected from students at baseline (pre-intervention) and examined as factors associated with a positive program evaluation. A positive evaluation was defined as students selecting one of the top two response options (e.g. “Completely relevant” or “Somewhat relevant”) on a 5-point Likert scale when rating key aspects of the program, including enjoyment, relevance, and perceived utility.

*Gender identity*: measured using an item extracted from surveys administered by ACON (an Australian LGBTQIA + affirmative organization). Responses included “male,” “female,” “non-binary/gender fluid,” “other identity,” and “prefer not to say”.

*Primary language spoken at home*: English or other.

*Relative socioeconomic disadvantage*: Family Affluence Scale III (FAS; low, moderate and high), which has been validated among adolescents [17].

*Geographical location*: metropolitan or regional, based on the schools’ geographical remoteness classification as per the Australian Statistical Geography Standard Remoteness Structure [18].

*School grades:* “What grades do you usually get in school.” Responses were made on a 6-item scale: “90%–100%,” “80%–89%,” “70%–79%,” “60%–69%,” “50%–59%,” and “49% and below.”

*Truancy*: “How many days did you have off school last year without your parents’ permission?”. Response options were on a 5-item scale: “0 days,” “1–2 days,” “3–5 days,” “6–10 days,” “11+ days”.

*Knowledge about the Big 6*: 20-item scale, developed to assess knowledge of Australian guidelines in relation to physical activity, screen time, sleep, alcohol, and dietary intake; prevalence of alcohol and tobacco use; and physical and mental health effects of the Big 6. For each statement, students answered “True,” “False,” or “Don’t Know.” Scores were summed for a total knowledge score.

*Self-efficacy*: General Self Efficacy Scale [19]. Ten items (e.g. “I can usually handle whatever comes my way”) measured on a 4-point scale ranging from 1 (not at all true) to 4 (exactly true).

*Self-control:* Brief Self-Control Scale [20] adapted for adolescents.

### Contextual factors

Barriers and facilitators to successful implementation were assessed via student and teacher evaluation surveys and fidelity logbooks. Teachers were asked to list “ways in which the *Health4Life* program could be improved in the future” and to make additional open-ended comments regarding the *Health4Life* modules. Students were also asked to name “one good thing” and “one bad thing” about the program in two open-ended, qualitative questions (see Supplementary Material for full surveys).

### Analysis

Descriptive statistics described quantitative implementation and acceptability data.

To identify factors associated with a positive student evaluation of *Health4Life*, we conducted mixed effects logistic regressions, with a random effect for school included to account for the clustered nature of the study design and data. We focused analysis on three items; (i) Learning style (“*How much did you like learning in this way (i.e. via online cartoon modules)?*”), (ii) Storyline relevancy (“*How relevant were the stories to experiences in your own life?*”), and (iii) Future use of skills and information (“*How likely are you to use the skills and information taught in the program in your own life?*”). Unadjusted bivariate associations were tested first in separate regression models for each of the quantitative outcomes and the baseline factors (e.g. association of gender with relevancy of the storyline). Next, all factors were entered into a multivariable model to determine which baseline factors were uniquely associated with a positive evaluation of the program. As we were testing multiple outcomes, we controlled the potential false positive rate by including a Bonferroni adjustment for significance (*α* 0.05/3 = 0.017). To assess potential bias due to missing data, logistic regression analyses were conducted to compare baseline demographic and individual characteristics between students who completed the evaluation survey to those who did not.

For qualitative data, two researchers analyzed open-text survey responses using an inductive, codebook thematic analysis approach in Microsoft Excel [21, 22]. This approach was chosen to systematically code and organize participant responses, while allowing flexibility in developing themes as the analysis progressed to describe the acceptability, barriers and facilitators of *Health4Life* among Australian adolescents and their teachers. The researchers first immersed themselves in the data, repeatedly reading all open-text responses to gain familiarity. Initial codes were generated to form a codebook, which was iteratively refined as themes were developed. Reflexivity was considered throughout the process via ongoing critical reflection on assumptions and interpretive decisions by the two researchers. As such codes and themes were continuously reviewed and adjusted in relation to the data. In cases where a researcher was uncertain, they discussed with the second researcher until they reached decision.

## Results

### Sample characteristics

A total of 118 teachers from 34 of 36 (94%) intervention schools completed the evaluation survey and 116 completed the fidelity logbook. Teachers were from Independent (46.6%), Government (39%) and Catholic schools (14.4%), and 19 schools were from the state of New South Wales (NSW) (54.3%), nine from Queensland (QLD) (25.7%) and six schools from Western Australia (WA) (20%).

Of the 3609 students in the intervention group, 2085 students (57.8%) from 33/36 intervention schools (91.6%) completed optional evaluation surveys [*M*_age_(SD) = 12.70 years (0.50), 51.4% male]. Most students spoke English as their first language at home (88.8%) and were from NSW (58.8%). Students were primarily from high (48.9%) or middle (33.6%) relative socioeconomic advantage. Most students reported no truant days (89.1%) and self-reported their usual grades as falling between 80% and 89% (34.6%) or 70% and 79% (27.5%). Their mean baseline knowledge score was 12 out of 20 (SD = 3). Attrition analyses revealed that higher knowledge (OR = 0.96, 95% CI = 0.93, 0.98) and higher self-control (OR = 0.98, 95% CI = 0.97, 0.99) at baseline was associated with lower odds of missing evaluation survey. Lower grades at baseline were associated with higher odds of not completing the evaluation survey compared to higher grades (OR = 2.84, 95% CI = 1.76, 4.60). Students of middle socioeconomic advantage, compared to low (OR = 1.28, 95% CI = 1.01, 1.63), and those who lived in regional locations, compared to metropolitan, (OR = 9.44, 95% CI = 1.98, 45.10, *P* = .005) had higher odds of not completing the evaluation survey. Of the three schools where students did not provide any evaluation data, two were in NSW and one was in QLD and all three were co-educational and in regional locations.

### RQ1: To what extent was the Health4Life intervention delivered and received as intended?

Teachers (*n* = 116) from 34/36 (91.6%) intervention schools provided fidelity data. Nearly all teachers (range: 96%–99% across modules) indicated that their students completed the online cartoon component for each of the six lessons. Additionally, most teachers reported that their students completed the whole online cartoon for each of the six modules (Module 1: 94%; Module 2: 96%; Module 3: 93%; Module 4: 90%; Module 5: 90%; Module 6: 89%). The most common reasons for incomplete delivery were a lack of time and technological issues.

Of 3157 intervention students with accurate objective module completion data, 1960 (62.1%) received a full dose (completed all six modules) and 1197 (37.9%) received an insufficient dose (<6 modules). As previously reported, a-priori exploratory analyses examined the effect of intervention dose on student outcomes [12]. These analyses showed no differences in primary risk behavior outcomes between students who received all six online modules (62.1%) and those in the control group. However, both full and insufficient doses (completed < 6 modules) of *Health4Life* were associated with greater knowledge of the Big 6 over time. Exposure to other program components (e.g. student and teacher summaries) was low-to-moderate (see Supplementary Table S.3).

### RQ2: How did participants (students and teachers) respond?

#### Teacher acceptability

Teachers (*n* = 118) from 34/36 (91.6%) intervention schools completed the teacher evaluation survey either online or hard copy. Overall, most teachers who completed the evaluation survey rated *Health4Life* favorably (84%; 99/118) and thought that *Health4Life* was better/much better than other school-based health education programs (69%; 81/118). Three-quarters (75%; 88/118) found the online component easy to implement and found it easy to access computer facilities (76%; 90/118). Most (71%; 83/118) thought the cartoon stories held the students’ attention well, students could recall the information after the cartoons well/very well (72%; 85/118) and students enjoyed the cartoon-based stories (77%; 100/118). Educational quality of the classroom activities was rated positively (83%; 98/118) and most teachers (81%, 95/118) believed the activities reinforced the information well. Finally, more than half (63%, 74/118) were likely to use *Health4Life* as a teaching resource in the future and would recommend the program to others (64%; 75/118).

#### Student acceptability

Of the 2085/3609 (57.8%) of intervention students who completed the evaluation, most (72.5% 1511/2085) rated *Health4Life* positively overall. Three quarters of students liked the cartoon stories (72.5% 1511/2085) and enjoyed the learning style (72.5% 1511/2085). Most (73.4% 1531/2085) found the information helpful and felt the skills and information would help them be healthy in the future (85% 1773/2085). Most students (67.6% 1410/2085) reported they were likely to use the skills and information from the program in their own lives. Half of the students (54.1% 1128/2085) felt that stories were relevant to their own lives and would recommend *Health4Life* to their friends (47.3%; 987/2085).

#### Factors associated with a positive student evaluation

Results from bivariate models examining factors associated positive with student evaluation of *Health4Life* are reported in the Supplementary Materials (Supplementary Table S.1). Results from the multivariable models are presented in Supplementary Table S.2. Identifying as female (OR = 1.47, 95% CI = 1.12, 1.92), having higher knowledge (OR = 1.11, 95% CI = 1.06, 1.15), and greater self-control (OR = 1.03, 95% CI = 1.02, 1.05) were associated with greater odds of reporting liking the learning style of *Health4Life*. Having greater baseline knowledge of the Big 6 was also associated with higher odds of finding the storylines relevant (OR = 1.06, 95% CI = 1.03, 1.10). Similarly, students who reported greater knowledge (OR = 1.1, 95% CI = 1.05, 1.14), higher self-control (OR = 1.05, 95% CI = 1.04, 1.07), and had higher socioeconomic advantage (OR = 1.74, 95% CI = 1.27, 2.38) had greater odds of being likely to use the skills/information in the future.

### RQ3: What contextual factors influenced implementation?

When asked to suggest improvements to *Health4Life*, teachers (*n* = 118) identified factors across three key areas: program content, website functionality and program delivery (Table 2). The most frequently cited areas for improvement included enhancing the in-class activities (27%, 32/118), followed by improving website functionality (25%, 30/118). Teacher suggestions primarily reflected barriers to implementation, including technological infrastructure (e.g. website speed), time constraints, and variability in student needs (e.g. varying literacy levels and engagement with content). Although no formal thematic analysis was conducted due to the small number of responses, fourteen teachers provided additional comments highlighting facilitators to implementation. These included consistent communication and support from the research team, the engaging and structured cartoon format, interactive features that supported student learning, and alignment with existing school infrastructure (e.g. students having their own devices).

**Table 2:** Summary of suggested teacher improvements to *Health4Life*.

Theme	Description	Example
Intervention design and content		
Improve in-class activities (*n* = 32)	More engaging activities, greater opportunities to check student recall and assess learning.	“More interactive activities to engage the students” (male teacher, NSW)
Increase accessibility (*n* = 28)	Incorporate audio voiceovers, video format, and reduce the number of cartoon slides.	“Voiceover options for low literacy students” (male teacher, NSW)
Improve module content (*n* = 24)	Reduce repetition, shorter modules and greater variety to ensure material is relevant and challenging.	“Sections of the student tasks were quite repetitive” (male teacher, NSW)
Improve student experience (*n* = 16)	Increase appeal of cartoon characters, ease of signing in, and more dynamic content to maintain student interest.	“Students didn’t really have any affection towards the characters” (male teacher, QLD)
Website functionality		
Improve user experience (*n* = 30)	Optimize the website to increase loading speed, allow progress-saving, fix glitches, and allow multiple students to access the site simultaneously.	“Loading times, make sure to improve as it makes a huge impact on engagement” (female teacher, WA)
Greater teacher control (*n* = 20)	Teachers wanted to monitor student progress, check the quality of student work, and to simplify the login process.	“Ability to track student completion of activities online would be beneficial” (male teacher, QLD)
Intervention delivery and compatibility		
Allocate more time (*n* = 9)	Greater time needs to be allocated to complete the modules, activities and to discuss learnings.	“We were told around 20 minutes per module, realistically it took around 40 minutes for our year 7 students” (female teacher, NSW)
Greater flexibility (*n* = 7)	Desire for flexible delivery methods to cater to specific class needs.	“Have a collaborative option for running programs so it can be worked through as a class. Some students that had lower levels of literacy struggled to get through the tasks” (male teacher, NSW)
No improvements		
“Nothing” to improve (*n* = 22)	Many teachers were satisfied with current program content and delivery	“I have no significant suggestions, it seemed to work well” (female teacher, NSW)

When asked to list “one bad thing about the *Health4Life* program,” 493/2085 (23.6%) student responses could not be coded due to being nonsensical. Thematic analysis of the 1592 valid responses found that comments relating to program content were commonly reported (443/1592, 27.8%), followed by reports that nothing was bad about the program (309/1592, 19.40%), and functionality issues when using the program (309/1592, 19.40%) (see Table 3). When asked to “list one good thing about the *Health4Life* program,” comments relating to program content and design were most frequently reported (1185/1970, 60.2%), followed by mode of delivery (522/1970, 26.5%) and relatability/relevance (206/1970, 10.5%; Table 4).

**Table 3:** Results from thematic analysis of student responses to “list one bad thing about *Health4Life*.”

Theme	Example
Intervention Content (*n* = 443)	
Relatability (*n* = 190)	“The cartoons are not relevant to the average life of teenage girls/boys” (female, age 12)
Storylines and characters (*n* = 57)	“The relationship between Seb and Anna wasn’t spicy enough” (female, age 12)
Nothing (*n* = 309)	“Nothing, its perfect!” (male, age 13)
Functionality issues (*n* = 309)	
Slow to load (*n* = 107)	“It took a while to load modules” (female, age 12)
Glitching (*n* = 81)	“I didn’t like it when the program glitched” (female, age 12)
Mode of delivery (*n* = 68)	“Characters should be able to speak with sound”—(female, age 12) “It doesn’t save…” (male, age 12)
Intervention length (*n* = 347)	
Program too long (*n* = 293)	“The cartoons are way too long” (male, age 13)
Program too short (*n* = 54)	“It was too short and now I want to know how everything ends” (male, age 13)
Everything (*n* = 36)	“Everything about the program” (male, age 13)

**Table 4:** Results from thematic analysis of “list one good thing about *Health4Life*.”

Theme	Example
Intervention content (*n* = 1185)	
Educational/informative (*n* = 484)	“I like the summaries because you could always look back and revise what you learnt” (female, age 13)
Helpful (*n* = 285)	“After doing this program, I have actually made changes in my own lifestyle. This makes me feel better about myself…” (female, age 13)
Storylines (*n* = 251)	“I really enjoyed the story and characters overall, and so do my classmates!” (male, age 13)
Interesting (*n* = 76)	“The cartoons were an interesting way to learn about being healthy that made me look forward to learning about my health…” (female, age 13)
Easy to understand (*n* = 74)	“Health4Life did an amazing job at explaining, it is an easy to understand way for kids…” (female, age 13)
Intervention delivery and learning style (*n* = 522)	
Cartoons (*n* = 256)	“I really liked how Health4Life included cartoons to show different situations, I also like the diversity” (female, age 12)
Fun (*n* = 189)	“It’s a fun and engaging way to learn lots of important information we are not just staring at a blank piece of text and spacing out” (female, age 13)
Interactivity (*n* = 55)	“The Health4Life program teaches kids in an interactive way with cartoons and short activities at the end” (female, age 12)
Online delivery (*n* = 22)	“It’s fun to do it on the computer” (male, age 13)
Relatability/relevance (*n* = 206)	
Relatability (*n* = 108)	“The scenarios really related to me and gave me a message if I was stuck in that situation” (female, age 13)
Relevance (*n* = 98)	“It was very relevant and it was something the class WANTED to do, not just another bit of school work” (male, age 13)
Nothing good (*n* = 33)	“Can’t think of a good thing” (male, age 12)
Other (*n* = 24)	“It got me off work” (male, age 13)

## Discussion

This study presents the findings from a process evaluation of the *Health4Life* school-based program. Overall, we found that teachers reported delivering *Health4Life* with high fidelity and while the program was largely well-received by teachers and students, areas for improvement were identified. Interpreted through the CFIR framework, our findings highlight how implementation was shaped by intervention characteristics and contextual factors in the school setting. Our findings have important implications for the design and delivery of future digital school-based interventions, particularly in terms of acceptability and equity. Key recommendations are discussed below and summarized in Table 5.

**Table 5:** Key recommendations for the design, implementation and process evaluation of future digital school-based interventions.

Design	(1) Engage in iterative co-design with young people from diverse backgrounds and schools to ensure relevance and inclusivity in content and delivery, and to ensure that program design does not inadvertently widen disparities in engagement or impact. (2) Develop tailored content based on factors such as gender or SES, e.g. using branching logic to guide users to storylines specific to their context/needs. (3) Integrate technology to enhance user acceptability and create more immersive learning experiences, e.g. audio, artificial intelligence, virtual reality. (4) Ensure web tools are responsive and fast loading, as website performance impacts student acceptability and program usability.
Implementation	(5) Develop strategies to address technological infrastructure barriers in schools, e.g. offering offline or low-bandwidth program options. (6) Offer structured teacher training before and throughout program implementation to support confident delivery and effective integration into classroom practice. (7) Design programs with flexible delivery options to accommodate varied learning needs and styles, e.g. allowing early finishers to begin additional activities while others complete core tasks.
Process evaluation	(8) Complement quantitative survey data with qualitative interviews to capture richer, more nuanced insights from students and teachers about program implementation. (9) Collect teacher-level data (e.g. motivation, confidence, attitudes) to better understand how these factors affect implementation and student outcomes. (10) Gather school-level and broader contextual data (e.g. school and departmental policies and infrastructure) to identify environmental factors that might influence program implementation and efficacy.

Given that Health4Life was delivered within the mandatory school health education curriculum, adoption of the intervention within participating schools was high by design. Teacher-reported fidelity was high for the online cartoon modules, with nearly all teachers indicating that their students completed each of the six lessons. However, objective dose data indicated that only 62% of students completed all six online modules. This discrepancy likely reflects the difference between class-level delivery and individual student exposure. While teachers may have reported that lessons were delivered as intended, objective data indicate variability in student-level completion, possibly due to factors such as student absence, incomplete engagement with the modules, or other individual differences in participation. However, objective data is unable to capture adaptations, deliberate or unintended, made by teachers during implementation. In fact, based on teacher report, while most students received *Health4Life* as prescribed (i.e. completed individually, online), a minority (12.5%) of students attended schools where the program was delivered differently. Some teachers anecdotally reported adapting the delivery format, choosing to print resources or facilitate the cartoon modules as a group or via role-play, to overcome implementation challenges and increase levels of engagement. These findings illustrate the interaction between intervention design and the school context, whereby the intended individualized digital delivery was influenced by classroom constraints and teaching practices, and highlight the role of adaptation in response to contextual challenges. Findings also support a multi-method approach to evaluating intervention implementation, including objective evidence and teacher self-report [23].

In this study, no formal teacher training was provided and the level of support provided prior to and during implementation varied. Typically, support included an initial meeting with relevant school staff and a research assistant, and access to implementation guidelines. Some schools opted for an induction session with all staff administering the program, whilst others preferred to receive information via email to distribute. Analysis of fidelity and dose data indicated that many teachers did not engage with the optional resources provided. For example, while half of the teachers accessed the accompanying teacher summary for the first module, this dropped to just 17% by the sixth module. Although not having to undergo intensive training is a strength of a program for time-poor teachers, research suggests that training is a key factor associated with implementation fidelity [24]. In addition to ensuring teachers are adequately prepared and confident to use the program in their class, training may also foster a commitment to the intervention and a greater understanding of the importance of intervention fidelity that can lead to improved student outcomes [1, 24, 25]. Future digital school-based interventions may benefit from offering more structured training to ensure they are sufficiently embedded into teaching practice and tailored to specific contexts and needs. Future studies should also assess teachers’ competence in implementing the intervention, alongside adherence, particularly when evaluating the impact of training and implementation support.

In terms of how participants responded to the intervention, most teachers rated the program favorably and were likely to recommend and use *Health4Life* in the future. Most teachers reported that the web-based delivery easy to implement and integrate within existing school infrastructure. According to teacher reports, the main external barriers to implementation were technical issues and a lack of time. While quantitative teacher evaluation data indicated that they valued the complementary classroom activities, the most common suggestion for improvement in the open-ended responses was to enhance these activities. This suggests that while teachers recognize the importance and potential of the classroom activities, there may be unmet expectations around their adaptability to different teaching contexts and different learning needs. Relatedly, a key suggestion for improvement was to include audio narration to support students with lower literacy and/or visual impairments, while enhancing overall student engagement. Students mostly provided positive feedback about *Health4Life*, finding it helpful, an enjoyable way to learn, and informative for improving their health behaviors. Although some students suggested improvements in relation to program content, length and website functionality, key strengths were the educational and informative nature of the content, and the fun and interactive delivery style. While 56% of students reported that the storylines were relevant to their own lives, responses to open-ended questions revealed contrasting views. Specifically, a lack of relatability emerged as a negative aspect of the program for some students, yet this was the best part of the program for others. This highlights that *Health4Life* resonated differently among students and suggests that better tailoring of content may be needed to reflect the diverse experiences of teenagers. These findings also align with CFIR constructs relating to the innovation itself (i.e. the intervention), particularly design and adaptability. Positive feedback from students and teachers on the cartoon format, interactivity, and mode of delivery highlights the importance of well-designed and engaging digital content, while suggestions to enhance classroom activities and tailor delivery reflect the need for adaptability to diverse classroom contexts. At the same time, reported technological issues and time constraints underscore the influence of inner setting factors, such as available resources and compatibility with existing school workflows, on implementation fidelity and student engagement.

Students with higher baseline knowledge of the Big 6 had greater odds of enjoying the learning style of *Health4Life*, finding the storylines more relevant, and reporting that they would use the skills and information in the future. It is possible that having a stronger initial understanding of some of the concepts covered in *Health4Life* enabled students to better understand and connect with the content, leading to a higher level of interest and enjoyment. In addition, we found that females had greater odds of enjoying learning via the online cartoons. Although we did not conduct qualitative interviews for this study, further consultation with students would be useful to identify alternative ways to engage young males in *Health4Life*, such as gamification or more immersive learning styles. Students experiencing greater socioeconomic advantage were more likely to report that they would use the skills and information in *Health4Life* in the future, which aligns with a recent analysis of the implementation of the program specifically among students experiencing disadvantage [26]. The less favorable ratings among students experiencing disadvantage may be due to the fact that *Health4Life* was co-designed only with students from independent schools, and more advantaged backgrounds [9]. Together, these findings highlight the importance of iterative co-design with diverse groups of youth and tailoring the content and delivery of digital interventions by gender, socio-economic status, and knowledge levels to maximize acceptability and effectiveness. This aligns with previous implementation research in schools suggesting that interventions are more likely to be acceptable and feasible when they are developed collaboratively with end users, and when sufficient flexibility is built into content and delivery to meet the needs of different students[27]. From an equity-focused implementation perspective, these differences also highlight the need to ensure interventions are designed and delivered in ways that do not inadvertently widen disparities in engagement or impact, recognizing that implementation cannot be considered successful unless it is equitable [15, 28].

Overall, our findings suggest that several implementation factors (e.g. technological barriers, variability in delivery, and contextual constraints) influenced participant acceptability and program implementation. These findings align with broader research demonstrating that time constraints, resource limitations, logistical challenges, and school contextual factors are among the most common influences on implementation of school-based prevention interventions [8, 27]. As such, a Type III error cannot be ruled out, whereby limitations in implementation may have contributed to the lack of observed behavioral effects. However, as reported in the original RCT [12], multiple factors likely contributed to the null effects, including the limited impact of education alone on behavior change, the absence of more intensive or multilevel strategies, the brief universal nature of the intervention, and broader contextual influences, such as the COVID-19 pandemic. Our findings also raise the possibility that achieving behavior change may require not only effective implementation, but also careful selection and optimization of behavior change techniques and intervention components. Recent evidence suggests that effective digital interventions for adolescent health behaviors commonly incorporate social support, particularly parental and peer involvement, self-monitoring, goal setting, and feedback [29]. Although Health4Life included several of these, self-monitoring and goal setting were primarily delivered through the companion smartphone app, which had very low uptake [11], and further adapting the web-based feedback to align with students’ readiness to change may have increased its relevance and behavioral impact. While the original school-based program did not include parental support, an optimized version of Health4Life is currently being evaluated to determine whether the addition of a companion parent component leads to stronger behavioral outcomes [30]. Importantly, the knowledge gains and improvements in student wellbeing observed in the Health4Life RCT [16, 25] have inherent value in school settings, where improving health literacy and supporting student wellbeing are key educational priorities. As such, Health4Life is currently available to Australian schools via the OurFutures Institute, a not-for-profit organization. This process evaluation therefore plays a critical role in informing ongoing optimization of program delivery, with the aim of strengthening implementation, increasing engagement, and supporting sustained delivery in real-world school contexts, which is consistent with CFIR’s focus on sustainment of interventions [31].

### Limitations

Firstly, there was potential selection bias in the evaluation data. Only half of all eligible teachers and 58% of eligible students completed the evaluation surveys, and three entire schools did not provide any evaluation data. Missing analyses showed that students who were from metropolitan areas, had higher baseline grades, knowledge and self-control were more likely to complete the evaluation survey. This suggests that respondents may have been more advantaged or motivated than non-respondents. The acceptability findings may therefore disproportionately reflect the views of students who were better positioned to engage with the intervention, potentially leading to overestimation of acceptability and limiting generalizability to more disadvantaged students. Secondly, some data were only assessed at the class- or teacher-level, rather than the individual student-level, so we were unable to link all implementation outcomes to students’ evaluations, nor did we assess whether more favorable student evaluations were associated with changes in the trial’s primary risk behavior outcomes. Thirdly, it is possible that teacher-reported fidelity may have been influenced by social desirability bias. In addition, we did not systematically collect data on several important determinants of implementation, including key CFIR constructs related to the roles and characteristics of individuals (e.g. teachers’ motivation and capability to deliver the intervention) and the inner setting (e.g. school culture, communication, mission alignment). We also did not assess broader outer setting influences, such as factors related to state education systems and policy environments [15]. This limits our ability to fully understand determinants of implementation across participating schools. It is also important to acknowledge that this study was conducted in the Australian context, which may limit the generalizability of some findings. In Australia, health education is a mandatory subject until Year 10 (∼age 16), requiring delivery of curriculum content covering the Big 6, and it is possible that implementation may be more challenging in contexts without dedicated time for these topics. However, previous international studies have identified similar implementation barriers and facilitators for school-based health interventions [32]. Moreover, interventions built on the *OurFutures* prevention model, which underpins *Health4Life*, have demonstrated acceptability, feasibility, and usability among students and teachers in international contexts [33, 34] which support the generalizability of the current findings beyond the Australian context. Finally, although two researchers were involved in coding and theme development of qualitative data, the data were not double-coded. Independent double-coding may have facilitated the exploration of alternative interpretations and the identification of additional or secondary themes [35].

## Conclusion

The *Health4Life* school-based digital intervention was rated favorably by students and teachers, indicating that an online cartoon-based peer-to-peer learning program is a well-received approach for delivering health promotion within schools. This process evaluation highlights the importance of flexibility in digital program design and delivery, particularly the ability to tailor content to ensure it resonates with students from diverse backgrounds. Key areas for refinement include resolving technological issues to ensure a more seamless user experience and adapting the cartoon storylines to better reflect the lived experiences of a broader student population. Although this study had a moderate response rate, the findings nonetheless offer valuable insights for the ongoing refinement of *Health4Life*, as well as informing best practices for future school-based digital interventions.

## Funding sources

The Health4Life Study was funded by the Paul Ramsay Foundation and the Australian National Health and Medical Research Council. K.E.C. was funded by a University of Sydney Horizon Fellowship. L.A.G. [#2033054], C.C. [#2026552], and M.T. [#1195284] were funded by NHMRC Investigator grants. L.T. was funded by a grant from the Medical Research Future Fund [MRF2017307]. B.S. was funded by U.S. National Institutes of Health R01 grant (DK125404).

## Role of the funder

The funders had no role in the design and conduct of the study; collection, management, analysis, and interpretation of the data; preparation, review, or approval of the manuscript; or decision to submit the manuscript for publication.

## Supplementary Material

ibag060_Supplementary_Data

## Data Availability

De-identified data from this study are not available in a public archive. Requests for de-identified data from this study will be considered (as allowable according to institutional ethics committee standards) by emailing the corresponding author.

## Welfare of animals

This article does not contain any studies with animals performed by any of the authors.
