# Supplementary material for: Process evaluation of a digital school-based intervention: lessons learned from the Health4Life study
Source: Transl Behav Med. 2026 Sep 14;16(1):ibag060. doi: 10.1093/tbm/ibag060 (PMC13573580; doi:10.1093/tbm/ibag060)
Supplement: Supplementary file 1 [file ibag060_supplementary_data.docx]

**Supplementary Materials**

Table of Contents

[1.	Student Evaluation Survey	2](#_Toc229053926)

[2.	Teacher Evaluation Survey	4](#_Toc229053927)

[3.	Example Teacher Logbook	7](#_Toc229053928)

[4.	Results: Bivariate models of associations between predictors and positive evaluations	8](#_Toc229053929)

[5.	Table S.1 Summary table of bivariate mixed effects logistic regression models investigating associations between predictors and positive student evaluation	10](#_Toc229053930)

[6.	Table S.2 Summary table of multivariable mixed effects logistic regression models investigating associations between predictors and positive student evaluation	12](#_Toc229053931)

[7.	Table S.3: Exposure to the supplementary *Health4Life* components	14](#_Toc229053932)

[8.	Health4Life Logic Model	16](#_Toc229053933)

# 1. Student Evaluation Survey

**Health4Life Program – Online Student Evaluation**

Thank you for completing the *Health4Life program*. We would now like to get your feedback about the program. Please answer the questions below.

***The questions below refer to the online cartoon modules***

1. **Overall, how would you rate the Health4Life program?**

**☐** Very Good

**☐** Good

**☐** Average

**☐** Poor

**☐** Very Poor

2. **How much did you like learning in this way (i.e. via online cartoon modules)?**

**☐** Liked a lot

**☐** Liked a little

**☐** Neither liked nor disliked

**☐** Disliked a little

**☐** Disliked a lot

3. **How much did you like the stories in the Health4Life cartoons?**

**☐** Liked a lot

**☐** Liked a little

**☐** Neither liked nor disliked

**☐** Disliked a little

**☐** Disliked a lot

4. **How relevant were the stories to experiences in your own life?**

**☐** Completely relevant

**☐** Somewhat relevant

**☐** Unsure

**☐** Somewhat irrelevant

**☐** Completely irrelevant

5. **How helpful was the information to you?**

**☐** Extremely helpful

**☐** Somewhat helpful

**☐** Neither helpful or unhelpful

**☐** Somewhat unhelpful

**☐** Extremely unhelpful

6. **Do you think the skills and information you received in the program will help you to be healthy in the future?**

**☐** Yes, I think they will help a great deal

**☐** Yes, I think they will help somewhat

☐ No, I don’t think they will help at all

☐ I’m not sure whether they will help or not

7. **How likely are you to use the skills and information taught in the program in your own life?**

**☐** Very Likely **☐** Likely **☐** Unsure **☐** Unlikely **☐** Very Unlikely

8. **Would you recommend the Health4Life program to your friends?**

**☐** Yes **☐** No **☐** Maybe

9. **Please list ONE good thing about the Health4Life program:**

10. **Please list ONE bad thing about the Health4Life program:**

# 2. Teacher Evaluation Survey

**Health4Life Program - Teacher Evaluation**

Thank you for completing the *Health4Life program*. We would now like to get your feedback about the program. Please answer the questions below.

1. **Overall, how would you rate the Health4Life program?**

**☐** Very good

**☐** Good

**☐** Average

**☐** Poor

**☐** Very poor

2. **How would you rate the Health4Life program in comparison to other school-based health education programs?**

**☐** Much better than most programs

**☐** Better than most programs

**☐** The same as most programs

**☐** Worse than most programs

**☐** A lot worse than most programs

3. **How easy did you find it to implement the Internet-based component of Health4Life program?**

**☐** Very easy

**☐** Easy

**☐** Average

**☐** Difficult

**☐** Very difficult

4. **How easy was it for you to gain access to the computer facilities at your school for the Health and Physical Education classes?**

**☐** Very easy

**☐** Easy

**☐** Average

**☐** Difficult

**☐** Very difficult

5. **How well do you think the cartoon stories held the students’ attention?**

**☐** Very well

**☐** Well

**☐** Average

**☐** Poorly

**☐** Very poorly

6. **How well do you think the students could recall the health information after the cartoon-based stories?**

**☐** Very well

**☐** Well

**☐** Average

**☐** Poorly

**☐** Very poorly

7. **How much do you think the students liked the cartoon-based stories?**

**☐** Liked a lot

**☐** Liked a little

**☐** Average

**☐** Disliked a little

**☐** Disliked a lot

8. **How would you rate the educational quality of the additional classroom activities included in the online Teacher Centre?**

**☐** Very good

**☐** Good

**☐** Average

**☐** Poor

**☐** Very poor

9. **How easy did you find it to use the online Teacher Centre to prepare the activities for your class lessons?**

**☐** Very easy

**☐** Easy

**☐** Average

**☐** Difficult

**☐** Very difficult

10. **How well do you believe the additional classroom activities helped to reinforce the health information to students?**

**☐** Very well

**☐** Well

**☐** Average

**☐** Poorly

**☐** Very poorly

11. **How likely would you be to use the Health4Life course as a teaching resource in the future?**

**☐** Very likely

**☐** Likely

**☐** Undecided

**☐** Unlikely

**☐** Very unlikely

12. **How likely is it that you would recommend the Health4Life course to others?**

**☐** Very likely

**☐** Likely

**☐** Undecided

**☐** Unlikely

**☐** Very unlikely

13. **Could you please list any ways in which you think the Health4Life program could be improved for the future?**

14. **Please list any additional comments you have regarding the Health4Life modules**

# 4. Example Teacher Logbook

| MODULE 1 | Yes | No |
| --- | --- | --- |
| Did your students complete the online *Health4Life* cartoon component for Module 1? | ☐ | ☐ |
| Did your students complete the *whole* online cartoon for Module 1?  *If not, please give reasons why:* | ☐ | ☐ |
| Did you go through the Student Summary with your class for Module 1, or instruct them to download it? | ☐ | ☐ |
| On average, how would you rate the level of engagement of your students with this module?   - Very engaged - Somewhat engaged - Neither engaged nor disengaged - Somewhat disengaged - Very disengaged | | |
| Which activities did your students complete?   - What’s in the fridge: online activity - The Health4Life app download (homework task, recommended) - Stop and Think: Healthy food choices - Question time, screen time - Sleep and me | | |
| Did you prompt students to download the Health4Life app during class time? *If not, please give reasons why (e.g. school policy about mobile phones, lack of time):* | ☐ | ☐ |
| Did you encourage or remind students to use the Health4Life app outside of class time? *If so, how?* | ☐ | ☐ |
| Do you have any comments / concerns about Module 1? (*Please explain)* | ☐ | ☐ |

# 5. Results: Bivariate models of associations between predictors and positive evaluations

Table S.1 gives a summary of the mixed effects logistic regression models investigating the bivariate associations between each student predictor and positive evaluations across three outcomes.

*Learning style:* In bivariate models of associations between predictors and positive evaluation of learning style (i.e., through cartoons), four predictors were positively associated with a favourable evaluation. Compared to identifying as male, identifying as female was associated with greater odds of enjoying the learning style (OR = 1.37, 95% CI = 1.07, 1.75, p = 0.013). Having higher baseline self-efficacy (OR = 1.03, 95% CI = 1.01,1.05, p < 0.001), self-control (OR = 1.04, 95% CI = 1.03, 1.05, p < 0.001), and knowledge (OR = 1.12, 95% CI = 1.08, 1.16, p < 0.001) were all associated with having greater odds of enjoying the learning style. However, reporting higher grades at baseline was negatively associated with positive evaluation of the learning style (OR = 0.89, 95% CI = 0.82, 0.96, p = 0.005).

*Storyline relevancy:* In bivariate models of associations between predictors and positive evaluation of storyline relevancy, two predictors were positively associated with greater odds of a favourable evaluation: having higher baseline self-efficacy (OR = 1.02, 95% CI = 1.01,1.04, p = 0.009) and baseline knowledge (OR = 1.07, 95% CI = 1.04, 1.10, p < 0.001). Reporting higher grades (OR = 0.91, 95% CI = 0.84, 0.98, p = 0.010), and higher truancy at baseline (OR = 0.85, 95% CI = 0.76, 0.95, p = 0.004) were associated with lower odds of positive evaluation of storyline relevancy.

*Future skill usefulness:* In bivariate models of associations between predictors and positive evaluation of future skill use, higher baseline self-efficacy (OR = 1.03, 95% CI = 1.01, 1.05, p < 0.001), self-control (OR = 1.05, 95% CI = 1.04, 1.07, p < 0.001), and knowledge (OR = 1.10, 95% CI = 1.07, 1.14, p < 0.001) were all associated with greater likelihood of using skills taught in the future. Additionally, relative to being from low socioeconomic advantage, being from high advantage was associated with greater odds of reporting using skills in the future (OR = 1.64, 95% CI = 1.25, 2.16, p < 0.001). Contrastingly, having higher grades at baseline was associated with lower odds of reporting being likely to use skills in the future (OR = 0.85, 95% CI = 0.78, 0.92, p < 0.001).

| 6. Table S.1 Summary table of bivariate mixed effects logistic regression models investigating associations between predictors and positive student evaluation | | | |
| --- | --- | --- | --- |
| Characteristic | Odds Ratio | 95% CI | p-value |
| **Learning style** | | | |
| Gender |  |  |  |
| Male (ref) | — | — | — |
| **Female** | **1.37** | **1.07, 1.75** | **0.013** |
| Gender diverse | 0.82 | 0.15, 4.61 | 0.819 |
| Prefer not say | 0.78 | 0.28, 2.15 | 0.633 |
| Language |  |  |  |
| English | — | — | — |
| Other | 1.06 | 0.73, 1.54 | 0.765 |
| Relative socioeconomic disadvantage‡ | | |  |
| Low (ref) | — | — | — |
| Middle | 1.09 | 0.80, 1.47 | 0.592 |
| High | 1.1 | 0.82, 1.48 | 0.522 |
| Remoteness |  |  |  |
| Major city | — | — | — |
| Inner or outer Regional† | 0.73 | 0.37, 1.41 | 0.348 |
| **Grades** | **0.89** | **0.82, 0.96** | **0.005** |
| Truancy | 0.97 | 0.86, 1.09 | 0.565 |
| **Self efficacy** | **1.03** | **1.01, 1.05** | **<0.001** |
| **Self control** | **1.04** | **1.03, 1.05** | **<0.001** |
| **Knowledge** | **1.12** | **1.08, 1.16** | **<0.001** |
| **Content relevancy** | | | |
| Gender |  |  |  |
| Male | — | — | — |
| Female | 0.95 | 0.77, 1.17 | 0.603 |
| Gender diverse | 0.86 | 0.17, 4.38 | 0.86 |
| Prefer not say | 0.4 | 0.15, 1.08 | 0.07 |
| Language |  |  |  |
| English | — | — | — |
| Other | 1.18 | 0.86, 1.61 | 0.302 |
| Relative socioeconomic disadvantage‡ | | |  |
| Low | — | — | — |
| Middle | 1.07 | 0.82, 1.40 | 0.621 |
| High | 1.2 | 0.93, 1.56 | 0.165 |
| Remoteness |  |  |  |
| Major city | — | — | — |
| Inner or outer Regional† | 0.88 | 0.52, 1.51 | 0.653 |
| **Grades** | **0.91** | **0.84, 0.98** | **0.01** |
| **Truancy** | **0.85** | **0.76, 0.95** | **0.004** |
| **Self efficacy** | **1.02** | **1.01, 1.04** | **0.009** |
| Self control | 1.01 | 1.00, 1.02 | 0.254 |
| **Knowledge** | **1.07** | **1.04, 1.10** | **<0.001** |
| **Use skills in the future** | | | |
| Gender |  |  |  |
| Male | — | — | — |
| Female | 1.1 | 0.87, 1.39 | 0.412 |
| Gender diverse | 0.44 | 0.09, 2.26 | 0.327 |
| Prefer not say | 0.35 | 0.14, 0.92 | 0.034 |
| Language |  |  |  |
| English | — | — | — |
| Other | 1.14 | 0.81, 1.60 | 0.463 |
| Relative socioeconomic disadvantage‡ | | |  |
| Low | — | — | — |
| Middle | 1.36 | 1.03, 1.80 | 0.03 |
| **High** | **1.64** | **1.25, 2.16** | **<0.001** |
| Remoteness |  |  |  |
| Major city | — | — | — |
| Inner or outer Regional† | 1.04 | 0.57, 1.92 | 0.892 |
| **Grades** | **0.85** | **0.78, 0.92** | **<0.001** |
| Truancy | 0.96 | 0.86, 1.08 | 0.525 |
| **Self-efficacy** | **1.03** | **1.01, 1.05** | **<0.001** |
| **Self-control** | **1.05** | **1.04, 1.07** | **<0.001** |
| **Knowledge** | **1.1** | **1.07, 1.14** | **<0.001** |
| *Note*. OR = Odds ratio; 95% CI = 95% confidence interval; bold text indicates significance at alpha 0.017. ‡ Family Affluence Scale (FAS) III. Scores were converted to ridit scores that compare socio-economic status with other people in the study sample, classified as lower (under 0.2), middle (0.2–0.59), or higher socio-economic status (0.6–1.0). † Australian Statistical Geography Standard. | | | |

# 7. Table S.2 Summary table of multivariable mixed effects logistic regression models investigating associations between predictors and positive student evaluation

| Characteristic | Odds Ratio | 95% CI | p-value |
| --- | --- | --- | --- |
| **Learning style** |  |  |  |
| Gender |  |  |  |
| Male (ref) | — | — |  |
| Female | 1.47 | 1.12, 1.92 | **0.005** |
| Gender diverse | 1.08 | 0.18, 6.30 | 0.933 |
| Prefer not say | 1.01 | 0.34, 3.00 | 0.992 |
| Language |  |  |  |
| English | — | — |  |
| Other | 1.01 | 0.66, 1.53 | 0.969 |
| Relative socioeconomic disadvantage | |  |  |
| Low (ref) | — | — |  |
| Middle | 0.98 | 0.70, 1.38 | 0.918 |
| High | 0.96 | 0.69, 1.34 | 0.820 |
| Remoteness |  |  |  |
| Major city | — | — |  |
| Regional | 0.77 | 0.42, 1.42 | 0.409 |
| Grades | 0.97 | 0.87, 1.07 | 0.518 |
| Truancy | 1.02 | 0.89, 1.18 | 0.736 |
| Self-efficacy | 0.99 | 0.97, 1.02 | 0.568 |
| Self-control | 1.03 | 1.02, 1.05 | **<0.001** |
| Knowledge | 1.11 | 1.06, 1.15 | **<0.001** |
| **Content relevancy** |  |  |  |
| Gender |  |  |  |
| Male | — | — |  |
| Female | 0.90 | 0.71, 1.14 | 0.371 |
| Gender diverse | 0.99 | 0.19, 5.11 | 0.987 |
| Prefer not say | 0.49 | 0.16, 1.49 | 0.209 |
| Language |  |  |  |
| English | — | — |  |
| Other | 1.23 | 0.86, 1.75 | 0.251 |
| Relative socioeconomic disadvantage | |  |  |
| Low | — | — |  |
| Middle | 1.05 | 0.78, 1.41 | 0.752 |
| High | 1.2 | 0.89, 1.60 | 0.226 |
| Remoteness |  |  |  |
| Major city | — | — |  |
| Regional | 0.92 | 0.53, 1.61 | 0.767 |
| Grades | 0.97 | 0.88, 1.06 | 0.513 |
| Truancy | 0.86 | 0.76, 0.98 | 0.025 |
| Self-efficacy | 1 | 0.99, 1.02 | 0.653 |
| Self-control | 1 | 0.99, 1.01 | 0.892 |
| Knowledge | 1.06 | 1.03, 1.10 | **<0.001** |
| **Use skills in the future** |  |  |  |
| Gender |  |  |  |
| Male | — | — |  |
| Female | 1.04 | 0.80, 1.34 | 0.788 |
| Gender diverse | 0.66 | 0.12, 3.53 | 0.630 |
| Prefer not say | 0.63 | 0.22, 1.86 | 0.407 |
| Language |  |  |  |
| English | — | — |  |
| Other | 1.10 | 0.74, 1.63 | 0.634 |
| Relative socioeconomic disadvantage | |  |  |
| Low | — | — |  |
| Middle | 1.36 | 0.99, 1.86 | 0.060 |
| High | 1.74 | 1.27, 2.38 | **<0.001** |
| Remoteness |  |  |  |
| Major city | — | — |  |
| Regional | 1.01 | 0.56, 1.85 | 0.961 |
| Grades | 0.99 | 0.89, 1.09 | 0.827 |
| Truancy | 1.00 | 0.87, 1.14 | 0.955 |
| Self-efficacy | 0.99 | 0.97, 1.01 | 0.241 |
| Self-control | 1.05 | 1.04, 1.07 | **<0.001** |
| Knowledge | 1.1 | 1.05, 1.14 | **<0.001** |
| *Note*. OR = Odds ratio; 95% CI = 95% confidence interval; bold text indicates significance at alpha 0.017. | | | |

# 8. Table S.3: Exposure to the supplementary *Health4Life* components

| **Component** | **Aim** | **Delivery Method** | **% of teachers who accessed resource (n=210)** | **% students who accessed**  **(n=** **3609)** |
| --- | --- | --- | --- | --- |
| Module summaries for teachers and students | To reinforce key content taught within the cartoons, consisting of national prevalence data and guidelines and benefits and consequences of engaging in sufficient or insufficient amounts of each of the risk behaviours. | Interactive pdfs, downloadable via study website | Module 1: 51% (range: 1-9 times)  Module 2: 27% (range: 1-5 times)  Module 3: 22% (range 1-5 times)  Module 4: 19% (range: 1-4 times)  Module 5: 19% (range: 1-6 times)  Module 6: 17% (range: 1-4 times) | Module 1: 23% (range: 1-6 times)  Module 2: 18% (range: 1-5 times)  Module 3: 15% (range: 1-4 times)  Module 4: 11% (range: 1-6 times)  Module 5: 11% (range: 1-5 times)  Module 6: 11% (range: 1-7 times) |
| Teacher support documents | - Guidelines for implementing the program - 5 propositions from PDHPE syllabus - Alignment with state syllabus and national curriculum | Colourful, interactive pdfs, downloadable via study website | 23%      24%    NSW : 16%  WA: 18% QLD: 16% | n/a |
| Student Activities (offline) | Each module included 4-5 activities, such as worksheets, class discussions, and quizzes, to develop self-management and interpersonal skills. | Colourful and interactive pdfs downloadable via study website. To be completed after the cartoon modules | On average, teachers reported facilitating 2 activities per module. | Module 1: 44%  Module 2: 30%  Module 3: 33%  Module 4: 24%  Module 5: 29%  Module 6: 23%  Summative assessment: 22% |
| Student Activities (online) | Each module included 1 web-based activity, such as an interactive worksheet, game, or quiz to provide an engaging and immersive learning experience. | Online via study website. To be completed after the cartoon modules. |  | Module 2: 71.5%.  Module 4: 69.4%^1^ |

^1^ Data not available for other modules

# 9. Health4Life Logic Model
